# Prognostication of progressive pulmonary fibrosis in connective tissue disease-associated interstitial lung diseases: A cohort study

**DOI:** 10.3389/fmed.2023.1106560

**Published:** 2023-02-27

**Authors:** Yu-Hsiang Chiu, Maaike F. M. Koops, Mareye Voortman, H. Wouter van Es, Lucianne C. M. Langezaal, Paco M. J. Welsing, Anna Jamnitski, Anne E. Wind, Jacob M. van Laar, Jan C. Grutters, Julia Spierings

**Affiliations:** ^1^Department of Rheumatology and Clinical Immunology, University Medical Center Utrecht, Utrecht, Netherlands; ^2^Division of Rheumatology, Immunology and Allergy, Department of Medicine, Tri-Service General Hospital, National Defense Medical Center, Taipei, Taiwan; ^3^Department of Pulmonology, University Medical Center Utrecht, Utrecht, Netherlands; ^4^Department of Radiology, St. Antonius Hospital, Nieuwegein, Netherlands; ^5^Department of Rheumatology, St. Antonius Hospital, Nieuwegein, Netherlands; ^6^Department of Pulmonology, ILD Center of Excellence, St. Antonius Hospital, Nieuwegein, Netherlands

**Keywords:** interstitial lung diseases, connective tissue diseases, pulmonary fibrosis, outcome predictors, immune-mediated inflammatory diseases

## Abstract

**Background:**

Connective tissue diseases-associated interstitial lung disease (CTD-ILD) is a heterogeneous condition that impairs quality of life and is associated with premature death. Progressive pulmonary fibrosis (PPF) has been identified as an important risk factor for poor prognosis. However, different criteria for PPF are used in clinical studies, which may complicate comparison between trials and translation of study findings into clinical practice.

**Methods:**

This is a retrospective single center study in patients with CTD-ILD. The prognostic relevance of PPF definitions, including INBUILD, ATS/ERS/JRS/ALAT 2022, and simplified progressive fibrosing (simplified PF) criteria, were examined in this cohort and validated in the other reported Dutch CTD-ILD cohort.

**Results:**

A total of 230 patients with CTD-ILD were included and the median follow-up period was six (3—9) years. Mortality risk was independently associated with age (adjusted HR 1.07, *p* < 0.001), smoking history (adjusted HR 1.90, *p* = 0.045), extent of fibrosis on high-resolution computed tomography (HRCT) at baseline (adjusted HR 1.05, *p* = 0.018) and baseline DLCO (adjusted HR 0.97, *p* = 0.013). Patients with regular pulmonary function tests in the first 2 years (adjusted HR 0.42, *p* = 0.002) had a better survival. The prognostic relevance for survival was similar between the three PPF criteria in the two cohorts.

**Conclusion:**

Higher age, smoking, increased extent of fibrosis and low baseline DLCO were associated with poor prognosis, while regular pulmonary function evaluation was associated with better survival. The INBUILD, ATS/ERS/JRS/ALAT 2022, and simplified PF criteria revealed similar prognostication.

## Introduction

Connective tissue diseases (CTD) are characterized by dysregulation of the immune system resulting in inflammation and subsequent tissue damage followed by fibrosis. In CTDs with lung involvement, inflammation and/or fibrosis of pulmonary parenchyma leads to deterioration of lung function, cough and shortness of breath. Interstitial lung disease (ILD) occurs in approximately 15% of CTD patients, depending on the type of CTD, and is associated with high mortality and decreased quality of life (1).

The disease course of CTD-associated ILD (CTD-ILD) is heterogeneous. Therefore, clinical characteristics and risk factors for poor prognosis are crucial in managing patients with CTD-ILD. In previous studies, several biomarkers, fibrotic high-resolution computed tomography (HRCT) at baseline, senior age, smoking, steroid use and progressive pulmonary fibrosis have been identified as predictors of poor prognosis in CTD-ILD (2–4).

Particularly, rapid deterioration of respiratory symptoms, lung function and progressive fibrosis on HRCT are referred to as progressive fibrosing interstitial lung diseases or progressive pulmonary fibrosis (PPF) (3, 5–7). Identification of patients with PPF is crucial for clinical practice, as these patients have a poor prognosis and may benefit from antifibrotic drugs similar to patients with idiopathic pulmonary fibrosis (IPF) in randomized controlled trials (8, 9); however, the definition of PPF criteria differ between studies. Furthermore, the American Thoracic Society, European Respiratory Society, Japanese Respiratory Society, and Asociación Latinoamericana de Tórax (ATS/ERS/JRS/ALAT) defined scientific societies-approved criteria in the 2022 guideline (7). The variety in criteria complicates study comparison and clinical implication. In this study, we aimed to explore the prognostic relevance of the different PPF criteria in patients with CTD-ILD.

## Methods

### Study population

This is a single center retrospective cohort study performed at the ILD Center of Excellence, St. Antonius Hospital, Nieuwegein, Netherlands. Patients diagnosed with CTD-ILD or interstitial pneumonia with autoimmune features between 2005 and 2021 were included when at least a baseline HRCT was available (10–12). Baseline was defined as the time of ILD diagnosis. All patients were discussed in multidisciplinary team meetings. Clinical characteristics, laboratory results and pulmonary function tests (baseline, 6 months, 1 year, and 2 years) were retrieved from the electronic medical records. This study was approved by the Medical Research Ethics Committees United (MEC-U, number R05-08A) and all patients provided written informed consent.

### Pulmonary imaging

High-resolution computed tomography results were collected at baseline, 1 and 2 years. Baseline HRCT patterns were classified according to the classification for idiopathic interstitial pneumonia (13, 14), listing as consistent with usual interstitial pneumonia (UIP), probable UIP, alternative diagnosis or indeterminate for UIP. Probable and consistent with UIP were summarized as UIP. The alternative diagnosis was then classified as non-specific interstitial pneumonia [NSIP, including fibrotic, cellular, or mixed (15)], lymphocytic interstitial pneumonia (LIP), organizing pneumonia (OP), desquamative interstitial pneumonia, nodular lymphocytic hyperplasia, pleuroparenchymal fibro-elastosis and acute interstitial pneumonitis (AIP). The predominant HRCT features were categorized into fibrotic, including features as reticulation and honeycombing, or inflammatory, including ground-glass opacity and consolidation (3, 16–18). The changes in fibrosis and inflammation over time were classified as progression, stable, or regression. Extent of fibrosis on HRCT was evaluated at all time points. HRCTs were evaluated by two experienced thoracic radiologists who were blinded to clinical information and pathology diagnosis.

### Criteria for progression

The INBUILD criteria included patients with ≥10% relative decline in percentage of predicted forced vital capacity (FVC), ≥5 and <10% relative decline in FVC with progressive fibrosis on HRCT or worsening of respiratory symptoms, or deterioration of both HRCT fibrosis and respiratory symptoms within 2 years despite standard (anti-inflammatory) treatment (8). The ATS/ERS/JRS/ALAT 2022 criteria were met with at least two of the following criteria; worsening of respiratory symptoms, fibrotic progression on HRCT and lung function deterioration [≥5% absolute decline in FVC and/or ≥10% absolute decline in percentage of predicted hemoglobin adjusted diffusing capacity of the lung for carbon monoxide (DLCO)] occurring within 1 year and without alternative explanation (7). The simplified progressive fibrosing (simplified PF) criteria were met with any of the following: ≥10% relative decline in FVC, ≥15% relative decline in DLCO, or progression of fibrosis on HRCT within 2 years [Supplementary Table S1; (3, 6)].

The prognostic relevance for mortality over time was evaluated for the INBUILD criteria, the ATS/ERS/JRS/ALAT 2022 criteria, and simplified PF criteria. The prognostic relevance of the three PPF criteria was then validated in a previously published Dutch CTD-ILD cohort at University Medical Center Utrecht (UMCU) (3).

### Statistical analysis

Categorical variables were presented in frequencies, and the difference between groups was examined in Fisher’s exact test. The distribution of the data was assessed in histograms. The continuous variables were presented in medians (interquartile range, IQR), and the difference between groups was determined using the Wilcoxon rank sum test. The hazard ratios (HR) for mortality risks were calculated using Cox regression, and variables with a value of *p* < 0.1 were included in a multivariable analysis with age, gender, smoking, comorbidities, and underlying CTD. The prognostic relevance for mortality and the PPF criteria was examined in the time-dependent receiver operator characteristic (ROC) model and visualized in area under curve (AUC) over time. Risk factors for PPF were examined in logistic regression. Missing data were omitted from each regression analysis. A value of *p* < 0.05 was considered statistically significant. All statistical analyses were performed using R 4.0.3.

## Results

### Patient characteristics

A total of 230 patients were included in this cohort, of which 122 (53%) were female. The median age was 63 (IQR 54—69) years. The median follow-up period was 6 (3—9) years. The underlying CTD diagnosis included rheumatoid arthritis (RA) in 77 patients (33%), idiopathic inflammatory myopathies (IIM) in 38 (17%), primary Sjögren’s syndrome (pSS) in 33 (14%), undifferentiated connective tissue disease (UCTD) in 32 (14%), systemic sclerosis (SSc) in 24 (10%), mixed connective tissue disease (MCTD) in eight (3%), systemic lupus erythematosus (SLE) in eight (3%), overlap syndrome in six (3%), spondyloarthropathy in three (1%), and antineutrophil cytoplasmic antibody-associated vasculitis in one (0.4%). Patients with RA, including RA overlap syndromes, were older [median 65 (IQR 62—73) years] than non-RA patients [median 60 (50—67) years, *p* < 0.001]. A total of 133 (58%) patients were past smokers, and 13 (6%) patients were current smokers. The median tobacco exposure was 18 (10—30) pack-years at baseline. In 104 (45%) patients, the diagnosis of CTD and ILD occurred within 6 months of one another. ILD was diagnosed in 100 (43%) patients with pre-existing CTD for more than 6 months, and the median CTD duration at ILD diagnosis was 6 (IQR 2—13) years. Twenty-six (11%) patients were diagnosed with CTD more than 6 months after ILD diagnosis. Antinuclear antibodies were positive in 106 (46%) patients. Other autoantibodies were detected, including rheumatoid factor in 71 (31%) patients, anti-SSA in 70 (30%), anti-citrullinated peptide antibodies in 61 (27%), and anti-Jo-1 in 25 (11%). The median body mass index was 27 (IQR 24—30). The median Charlson’s comorbidity index was 3 (IQR 2—4), including 32 (14%) coronary artery disease, 23 (10%) diabetes mellitus, 14 (6%) chronic obstructive pulmonary disease, 12 (5%) cerebrovascular accident, 11 (5%) heart failure, eight (3%) pulmonary arterial hypertension, six (3%) peripheral vascular disease (PVD) and three (1%) chronic kidney disease. The most commonly used immunomodulators at baseline were corticosteroids in 165 (72%) patients, methotrexate in 64 (28%), azathioprine in 55 (24%), mycophenolate mofetil in 47 (20%), and hydroxychloroquine in 45 (20%). Four patients were on antifibrotics at baseline [nintedanib (*n* = 2) and pirfenidone (*n* = 2)] (Table 1).

**Table 1:** Patient characteristics.

Baseline characteristics	Patients
Age, median (IQR)	63 (54—69)
Gender (Female), *n* (%)	122 (53)
BMI, median (IQR)	27 (24—30)
Immunomodulators, *n* (%)	
Corticosteroids	165 (72)
Steroid dose (mg/day), median (IQR)	15 (5—30)
Azathioprine	55 (24)
Mycophenolate mofetil	47 (20)
Methotrexate	64 (28)
Leflunomide	12 (5)
Hydroxychloroquine	45 (20)
Cyclophosphamide	34 (15)
Sulfasalazine	12 (5)
Rituximab	22 (10)
Tumor necrosis factor inhibitors	29 (13)
Abatacept	3 (1)
Tocilizumab	3 (1)
Tofacitinib	1 (0.4)
Anti-fibrotics, *n* (%)	
Nintedanib	2 (1)
Pirfenidon	2 (1)
Charlson’s comorbidity index, median (IQR)	3 (2—4)
Autoantibodies, *n* (%)	
Antinuclear antibody	106 (46)
Rheumatoid factor	71 (31)
Anti-citrullinated peptide antibodies	61 (27)
Anti-dsDNA	5 (2)
Anti-SSA	70 (30)
Anti-SSB	16 (7)
Anti-U1-RNP	12 (5)
Anti-SM	5 (2)
Anti-SCL-70	12 (5)
Anti-RNA polymerase III	1 (0.4)
Anti-centromere	8 (3)
Anti-PM-SCL	8 (3)
Anti-Jo-1	25 (11)
Anti-PL12	7 (3)
Anti-Th/To	3 (1)
Anti-Ku	3 (1)
Anti-Ej	2 (1)
Anti-Oj	1 (0.4)
Anti-SAE	2 (1)
Anti-MDA5	1 (0.4)
Anti-TIF1γ	1 (0.4)
Anti-Mi2α	1 (0.4)
Anti-Mi2β	2 (1)
Anti-MPO	1 (0.4)
Anti-PR3	1 (0.4)
Anti-Cardiolipin IgG	1 (0.4)
Anti-Cardiolipin IgM	1 (0.4)
Anti-β2-glycoprotein IgG	2 (1)
Negative for autoantibodies, *n* (%)	21 (9)

IQR, interquartile range; BMI, body mass index.

### Radiology and pulmonary function progression

Various HRCT patterns were observed at baseline: UIP in 63 (27%, of whom 35 patients had RA) patients, fibrotic NSIP in 21 (9%), cellular NSIP in 25 (11%), mixed NSIP in 79 (34%), OP in 34 (15%), LIP in four (2%), AIP in one (0.4%), two (1%) combined OP and mixed NSIP, and one (0.4%) indeterminate. UIP patterns were observed in 35 (45%) RA patients, including RA overlap syndrome, and 28 (18%) in other CTD, *p* < 0.001. HRCT features were predominantly fibrotic in 117 (51%) patients and predominantly inflammatory in 113 (49%). The predominantly fibrotic HRCT consisted of 63 (100%) UIP, 31 (39%) mixed NSIP, 21 (100%) fibrotic NSIP, one LIP, and one indeterminate pattern. Patients with fibrotic HRCT patterns were older compared to patients with inflammatory patterns [respectively, 65 (IQR 60—74) and 59 (IQR 49—65) years old, *p* < 0.001]. Low extent of fibrosis [<20% (19)] on baseline HRCT occurred in 214 (93%) patients; in the predominant fibrosis group, 102 (87%) patients had low extent of fibrosis on HRCT at baseline. In patients with predominantly inflammatory patterns, 38 out of 68 patients (56%) had less inflammation at 1 year and 26 out of 47 patients (55%) at 2 years. HRCTs were unavailable in 95 patients at 1 year (50 in the predominantly fibrotic and 45 in the predominantly inflammatory group), and 144 patients at 2 years of follow-up (78 in the predominantly fibrotic and 66 in the predominantly inflammatory group).

**Figure 1:**
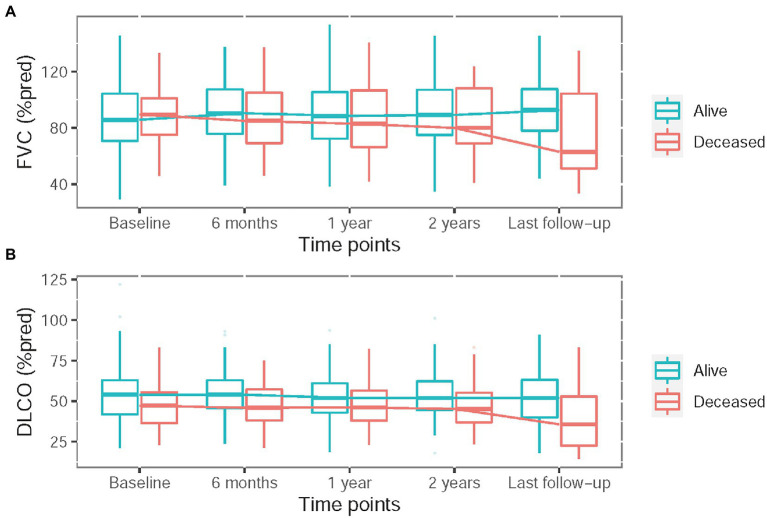
Serial change in pulmonary function test including percentage of predicted forced vital capacity (FVC) **(A)** and hemoglobin adjusted diffusing capacity of the lung for carbon monoxide (DLCO) **(B)**.

In the first 2 years, 112 (49%) patients had regular pulmonary function tests at 6 months, 1 year, and 2 years. The serial change in pulmonary function was shown in Figure 1. A relative decline ≥10% in FVC was seen in 22 (10%) patients at 6 months, 22 (10%) at 1 year, 32 (14%) at 2 years and 39 (17%) at the last follow-up. A relative decline ≥15% in DLCO was observed in 20 (9%) patients at 6 months, 28 (12%) at 1 year, 40 (17%) at 2 years and 40 (17%) at the last follow-up (Supplementary Figure S1).

Progressive pulmonary fibrosis in the first 2 years was observed in 61 (27%) patients meeting INBUILD criteria, 53 (23%) meeting ATS/ERS/JRS/ALAT criteria, 136 (59%) meeting simplified PF criteria and 125 (54%) when using simplified PF criteria with a threshold for HRCT ≥5% increase in the extent of fibrosis. The prevalence of PPF in each CTD was shown in Supplementary Table S2. Diagnosis of SSc, azathioprine use, PVD, regular follow-up pulmonary function, NSIP pattern and ANA positivity were revealed as predictors for more than two PPF criteria in univariable analysis; TNF inhibitor use was associated with reduced PPF risk. After multivariate adjustment, PVD and NSIP pattern remained significant as predictors for more than two PPF criteria (Supplementary Table S3). In RA patients, baseline HRCT with fibrotic NSIP pattern was associated with PPF meeting ATS/ERS/JRS/ALAT criteria (OR 6.04, *p* = 0.012) and INBUILD criteria (OR 7.60, *p* = 0.004). For other CTDs, no risk factors could be identified for more than two PPF criteria.

### Survival analysis

During follow-up, 68 (30%) patients died. The cause of death was ILD related in 17 (25%) patients, malignancy in nine (13%), COVID-19 in five (7%), other pulmonary infection in four (6%), heart failure in two (3%) and combined ILD and heart failure in four (6%), thrombosis in one (1%) and unknown in 26 (38%). Survival was independently associated with age (adjusted HR 1.07, *p* < 0.001), smoking history (adjusted HR 1.90, *p* = 0.045), and extent of fibrosis on HRCT at baseline (adjusted HR 1.05, *p* = 0.018). Higher baseline DLCO (adjusted HR 0.97, *p* = 0.013) and regular pulmonary function tests in the first 2 years (adjusted HR 0.42, *p* = 0.002) were associated with better survival (Table 2). In subgroup analysis, the association between UIP patterns and mortality was insignificant in RA patients (HR 1.3, *p* = 0.448) but significant in patients with other CTDs (adjusted HR 2.27, *p* = 0.030).

**Table 2:** Prognostic factors for survival.

Risk	HR	*p*-value	Adjusted HR	*P*-value
Male	2.19	0.002*	1.63	0.093
Age	1.08	<0.001*	1.07	<0.001*
Charlson’s score	1.47	<0.001*		
PAH	3.16	0.028*	2.29	0.118
Smoking history	2.46	0.002*	1.90	0.045*
RA	2.32	<0.001*	1.18	0.555
CTD duration	1.04	0.005*	1.02	0.232
TNFi	1.89	0.040*	1.71	0.142
Hospitalized infection	1.82	0.017*	1.25	0.405
Extent of fibrosis	1.03	0.019*	1.05	0.018*
DLCO	0.98	0.030*	0.97	0.013*
Regular PFT	0.399	<0.001*	0.42	0.002*
UIP	2.71	<0.001*	1.61	0.077
mNSIP	0.52	0.021*	0.60	0.078
OP	0.44	0.038*	0.53	0.129
NSIP	0.581	0.027*	0.78	0.327
Fibrotic patterns	2.56	<0.001*	1.64	0.094

PAH, pulmonary arterial hypertension; RA, rheumatoid arthritis; CTD, connective tissue disease; TNFi, tumor necrosis factor inhibitors; DLCO, percentage of predicted hemoglobin adjusted diffusing capacity of the lung for carbon monoxide; PFT, pulmonary function test; UIP, usual interstitial pneumonia; mNSIP, mixed non-specific interstitial pneumonia; OP, organizing pneumonia. *p < 0.05.

**Figure 2:**
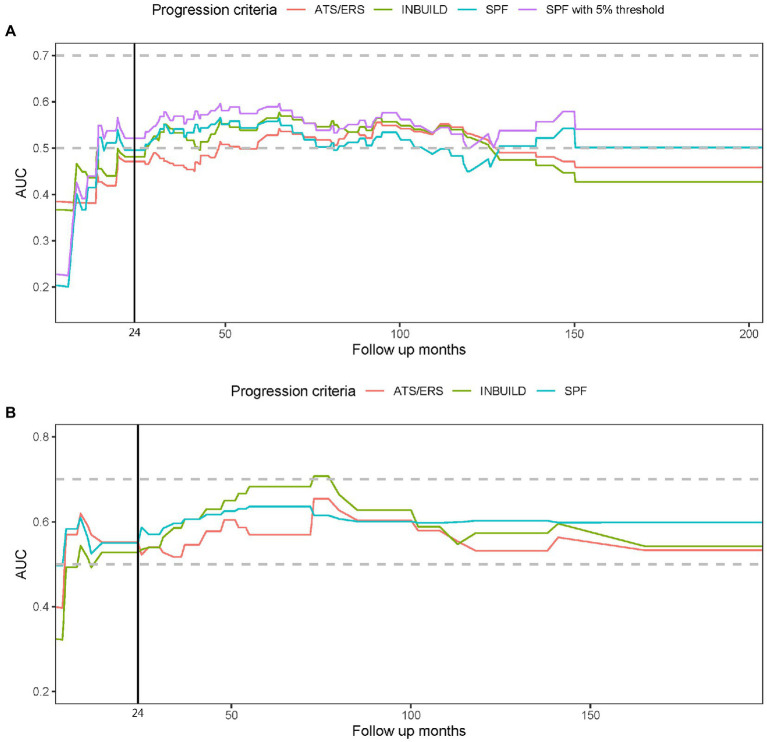
The prognostic relevance to mortality and progressive pulmonary fibrosis (PPF) is shown in this time dependent receiver operator characteristic (ROC) model. The figure demonstrates the area under the ROC curve (AUC) over the follow-up period in this cohort **(A)** and the validation cohort **(B)**. The vertical line indicates the timepoint of 24 months when PPF was identified. A higher AUC reflects a better correlation of the criteria with prognosis. The PPF criteria, including ATS/ERS/JRS/ALAT criteria (ATS/ERS), INBUILD criteria (INBUILD), and the simplified progressive fibrosing criteria (SPF), did not substantially outcompete each other. The prognostic value in AUC improved in SPF with defining HRCT progression with a ≥ 5% increase in fibrosis (SPF with 5% threshold) in the present cohort **(A)**.

None of the PPF criteria (in the first 2 years) achieved significant relation with mortality in Cox regression. The prognostic relevance did not differ between simplified PF criteria, INBUILD and ATS/ERS/JRS/ALAT criteria; the prognostic value improved in simplified PF criteria with defining HRCT progression with a ≥5% increase in fibrosis. The prognostic relevance of the PPF criteria with mortality risk over time in both cohorts is shown in Figure 2; The prognostic value of PPF criteria increased during the first 3 years and achieved a plateau thereafter in both cohorts.

## Discussion

This study explored the characteristics of patients with early CTD-ILD and their prognostic correlation with PPF. Increased age, smoking, and increased extent of fibrosis were associated with higher mortality risk, while higher baseline DLCO and regular pulmonary function tests were associated with reduced mortality risk. The prognostic relevance with mortality did not differ between simplified PF criteria, INBUILD and ATS/ERS/JRS/ALAT 2022 criteria.

The risk factors associated with mortality in this cohort are in line with identified risk factors in previous studies. Age and smoking are overarching risk factors across diseases (20). Patients with early diagnosis and subsequently low extent of fibrosis on HRCT and better DLCO, have a larger window of opportunity to initiate treatment in order to decrease the risk of progression. In addition, a large proportion of patients in this study had low extent of fibrosis at baseline, in contrast to previous studies, including the INBUILD trial and the validation cohort, in which more patients had high extent of fibrosis (3, 8). The correlation between mortality and PPF was also more prominent in patients with extensive lung fibrosis than in those with limited lung fibrosis in another SSc-ILD cohort (6).

In several studies, UIP pattern was observed more often in RA patients and was associated with mortality and DLCO decline (21, 22). In our study, RA patients were older and had UIP patterns more frequently than patients with other CTDs. However, this was not significantly associated with mortality. We did find an association with UIP pattern and mortality in the non-RA group. Similarly, in a recent RA-ILD study, UIP pattern was not associated with mortality or FVC decline at 2 years (23). A possible explanation is that treatment strategies in RA have improved tremendously in the last decades, whereas disease control in other underlying CTD diseases has proven more challenging. Moreover, not only UIP pattern was associated with predominant fibrosis; also, fibrotic NSIP and some other patterns could be linked to predominant fibrosis and were associated with increased risk for PPF. This finding is in line with the results of the validation cohort; predominantly fibrotic HRCT patterns revealed an increased risk for PPF (3, 18). Patients with predominantly inflammatory HRCT may respond better to anti-inflammatory treatment than those with predominantly fibrotic HRCT and therefore reduce the risk of PPF.

There may be a different risk profile of PPF in each CTD, while baseline severity, including lung function and HRCT, seems to be an overarching risk. In the European Scleroderma Trials and Research (EUSTAR) database, a large registry of SSc patients in Europe, male gender, higher modified Rodnan skin score and reflux/dysphagia symptoms were associated with FVC decline over 5 years in patients with SSc-ILD (24). In patients with RA-ILD, low baseline FVC/DLCO, UIP pattern, and steroid-use (>10 mg/day) were associated with progressive lung function decline (25). A positive serum anti-MDA5 is associated with rapid progression in IIM patients, but distinct clinical course was observed in subgroups (26, 27).

In recent years, PPF has received attention in trials increasingly, especially after the randomized trials with antifibrotic treatment. The natural history of PPF in ILD, including CTD-ILD, appears to be comparable with idiopathic pulmonary fibrosis (IPF) (28). Nevertheless, definitions of PPF vary across studies. The ATS/ERS/JRS/ALAT 2022 criteria were the first consensus of scientific societies but were based on data from IPF (7). As emphasized in the ATS/ERS/JRS/ALAT 2022 guideline, PPF should be utilized in prognostication instead of diagnosis. We examined the prognostic correlation of these PPF criteria in the time-dependent ROC model. The prognostic correlation with mortality was similar between the three PPF criteria and achieved a plateau after 3 years in this cohort (predominant CTD in RA) and the validation cohort (predominant CTD in SSc); the AUC in time-dependent ROC model was higher in the validation cohort than this cohort.

The strength of this study is that we validated the prognostication with two real world CTD-ILD data. The prognostic relevance was visualized in time dependent ROC model. Most patients were diagnosed early with low extent of fibrosis at baseline. However, the proportion of missing data was relatively high and can be regarded as limitation of this study (Supplementary Figure S1). As the St. Antonius Hospital is an ILD referral center, patients are often evaluated once for expert opinion after which follow-up will take place at local hospitals, which could largely explain the missing data at follow-up. In addition, patient reported respiratory symptoms were not systematically scored in the medical records, therefore we did not include this parameter in our analysis. In the validation cohort, 23 (15%) patients reported symptom progression from dyspnea on exertion to dyspnea at rest or oxygen requirement in the first 2 years. Because of the missing data at follow-up, the proportion of patients with PPF may be underestimated. Nonetheless, regular pulmonary function test in the first 2 years was associated with a significant preferable prognosis. A second limitation is that the reading of HRCT, which relies on experienced radiologists, may be variation in interobserver agreement, and radiological progression of most of the criteria is descriptive (3, 7–9, 29, 30). An artificial intelligence-aided quantitative HRCT evaluation could improve accurate detection of changes, although these techniques are not universally available yet (31, 32). Since CTD-ILD is a heterogenous manifestation, further research in biomarkers and artificial intelligence-aided HRCT analysis could support tailored clinical decision making.

In conclusion, we identified risk factors for mortality and examined prognostication of PPF in CTD-ILD patients. CTD-ILD is a rather heterogenous disease and the current PPF criteria may not be applicable universally. Disease control of the underlying CTD, multidisciplinary evaluation and systematic assessment of respiratory symptoms, pulmonary function, and HRCT are instrumental to identify high-risk patients and tailor treatment strategies (33). Further research is needed to explore optimal use of PPF criteria in managing patients with CTD-ILD.

## Data availability statement

The original contributions presented in the study are included in the article/Supplementary material, further inquiries can be directed to the corresponding author.

## Ethics statement

The studies involving human participants were reviewed and approved by Medical Research Ethics Committees United. The patients/participants provided their written informed consent to participate in this study.

## Author contributions

Y-HC, JL, JG, and JS conceptualized this study. MK and AW retrieved the clinical data. HE and LL analyzed the pulmonary images. Y-HC, MV, PW, AJ, JL, JG, and JS interpreted the clinical data. Y-HC, MK, and PW performed the formal analysis. Y-HC wrote the original draft. AW performed the data management. All authors have critically reviewed and agreed on all versions of the article, the article submission, and taking responsibility for all aspects of the work.

## Funding

This study was funded in part by a student grant from the government of Taiwan (Y-HC).

## Conflict of interest

JS and JL have received an unrestricted grant from Boehringer, JL has received honoraria from Abbvie, Arxx Tx, Galapagos, Gesyntha, Leadiant, Roche, and research grants from Astra Zeneca, MSD, and Roche.

The remaining authors declare that the research was conducted in the absence of any commercial or financial relationships that could be construed as a potential conflict of interest.

## Publisher’s note

All claims expressed in this article are solely those of the authors and do not necessarily represent those of their affiliated organizations, or those of the publisher, the editors and the reviewers. Any product that may be evaluated in this article, or claim that may be made by its manufacturer, is not guaranteed or endorsed by the publisher.
