# Supplementary material for: Prognostication of progressive pulmonary fibrosis in connective tissue disease-associated interstitial lung diseases: A cohort study
Source: Front Med (Lausanne). 2023 Feb 27;10:1106560. doi: 10.3389/fmed.2023.1106560 (PMC10008854; doi:10.3389/fmed.2023.1106560)
Supplement: Supplementary file 1 [file Data_Sheet_1.DOCX]

**Figure S1.**


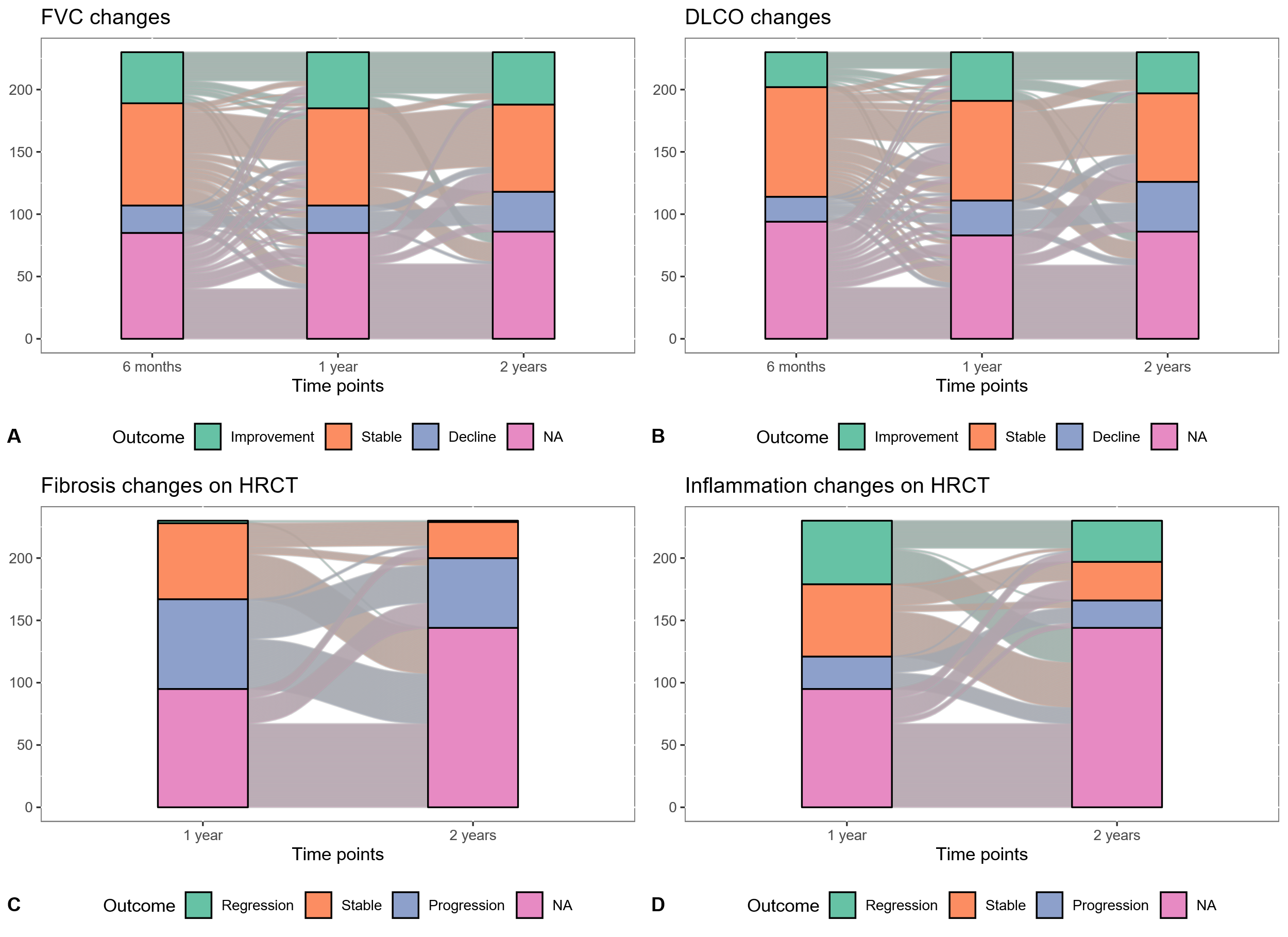


Figure S1. The alluvial plot shows the progress in forced vital capacity (FVC), diffusing capacity of the lung for carbon monoxide (DLCO), high-resolution computed tomography (HRCT) inflammation and HRCT fibrosis. Pulmonary function ≥ 10% in FVC and ≥ 15% relative change were defined as decline or improvement. NA, not available.

Table S1 Criteria for progressive pulmonary fibrosis

| Criteria names | Criteria definition |
| --- | --- |
| INBUILD criteria: any of the criteria within two years | ≥10% relative decline in FVC |
|  | ≥5% and <10 % relative decline in FVC with progressive fibrosis on HRCT or worsening of respiratory symptoms |
|  | Deterioration of both HRCT fibrosis and respiratory symptoms |
| ATS/ERS/JRS/ALAT 2022 criteria: at least two of the criteria within one year | Worsening of respiratory symptoms |
|  | Progression of fibrosis on HRCT:  a. Increased extent or severity of traction bronchiectasis and bronchiolectasis  b. New ground-glass opacity with traction bronchiectasis  c. New fine reticulation  d. Increased extent or increased coarseness of reticular abnormality  e. New or increased honeycombing  f. Increased lobar volume loss |
|  | Lung function deterioration:  ≥5% absolute decline in FVC and/or ≥10% absolute decline in DLCO |
| Simplified progressive fibrosing criteria: any of the criteria within two years | ≥10% relative decline in FVC |
|  | ≥15% relative decline in DLCO |
|  | Progression of fibrosis on HRCT |

Table S2. The prevalence of progressive pulmonary fibrosis (PPF) by ATS/ERS/JRS/ALAT 2022 criteria (ATS/ERS), INBUILD criteria (INBUILD), and the simplified progressive fibrosing criteria (simplified PF) in each CTD

|  | RA, n = 77 | IIM, n = 38 | pSS, n = 33 | UCTD, n = 32 | SSc, n = 24 | MCTD, n = 8 | SLE, n = 8 | Overlap, n = 6 | SpA, n = 3 | AAV, n = 1 |
| --- | --- | --- | --- | --- | --- | --- | --- | --- | --- | --- |
| ATS/ERS | 13  (17%) | 5  (13%) | 9  (27%) | 9  (28%) | 10  (42%) | 3  (38%) | 2  (25%) | 1  (17%) | 1  (33%) | 0 |
| INBUILD | 15  (19%) | 7  (18%) | 10  (30%) | 8  (25%) | 13  (50%) | 3  (38%) | 2  (25%) | 3  (50%) | 1  (33%) | 0 |
| Simplified PF | 42  (55%) | 24  (63%) | 23  (70%) | 19  (59%) | 14  (58%) | 5  (63%) | 4  (50%) | 3  (50%) | 2  (67%) | 0 |

Abbreviation: RA, rheumatoid arthritis; IIM, idiopathic inflammatory myopathies; pSS, primary Sjögren's syndrome; UCTD, undifferentiated connective tissue disease; SSc, systemic sclerosis; MCTD, mixed connective tissue disease; SLE, systemic lupus erythematosus; overlap, overlap syndrome; SpA, spondyloarthropathy; AAV, antineutrophil cytoplasmic antibody-associated vasculitis.

Table S3. Predictors for progressive pulmonary fibrosis

| Variable | ATS/ERS | | INBUILD | | Simplified PF | | Simplified PF + 5% | |
| --- | --- | --- | --- | --- | --- | --- | --- | --- |
|  | OR (95% CI) | aOR (95% CI) | OR (95% CI) | aOR (95% CI) | OR (95% CI) | aOR (95% CI) | OR (95% CI) | aOR (95% CI) |
| PVD | 7.14 (1.35 –52.63)* | 7.59 (1.29– 60.63)* | 5.86 (1.11– 43.08)* | 6.56 (1.17–50.35)* |  |  |  |  |
| DM | 2.38 (0.94– 5.80) | 2.59 (0.94– 6.94) | 2.35 (0.95– 5.68) | 2.41 (0.92–6.12) |  |  |  |  |
| BMI |  |  |  |  | 0.96 (0.90–1.01) | 0.95 (0.90–1.01) |  |  |
| RA | 0.55 (0.26– 1.07) | 0.70 (0.31– 1.50) |  |  |  |  |  |  |
| SSc | 2.31 (0.99– 5.22)* | 2.04 (0.82– 4.90) | 3.61 (1.62– 8.11)* | 3.67 (1.60–8.46)* |  |  |  |  |
| pSS |  |  |  |  |  |  | 2.14 (0.99–4.93) | 1.85 (0.83–4.37) |
| CTD duration |  |  |  |  | 1.00 (0.99–1.00) | 1.00 (0.99–1.00) | 1.00 (0.99–1.00)* | 1.00 (1.00–1.00) |
| Steroid use |  |  |  |  | 1.76 (0.98–3.15) | 1.44 (0.78–2.66) | 1.73 (0.97–3.10) | 1.43 (0.77–2.66) |
| Steroid dose |  |  |  |  | 0.98 (0.96–1.00) |  |  |  |
| AZA |  |  |  |  | 4.34 (1.59–15.24)* | 3.46 (1.23–12.37)* | 3.14 (1.28–8.89)* | 2.57 (1.01–7.44) |
| TNFi |  |  |  |  | 0.33 (0.13–0.79)* | 0.50 (0.17–1.39) | 0.26 (0.09–0.66)* | 0.41 (0.13–1.18) |
| Regular PFT | 3.15 (1.65– 6.21)* | 2.78 (1.41– 5.64)* | 2.33 (1.28–4.32)* | 2.12 (4.03–0.019) |  |  | 1.77 (1.05–3.02)* | 1.60 (0.93–2.77) |
| FVC |  |  |  |  | 0.99 (0.97–1.00)* | 0.99 (0.98–1.01) |  |  |
| UIP | 0.46 (0.20– 0.98) | 0.48 (0.19–1.11) |  |  | 0.52 (0.29–0.93) | 0.6 (0.32–1.14) |  |  |
| NSIP | 2.81 (1.46–5.71)* | 2.50 (1.24–5.29)* | 1.79 (0.99– 3.34) | 1.58 (0.83–3.05) | 2.06 (1.21–3.54)* | 2.5 (1.40–4.54)* | 1.76 (1.05–3.00)* | 1.79 (1.02–3.17)* |
| ANA | 1.91 (1.03– 3.61)* | 1.43 (0.67–3.09) | 2.04 (1.13– 3.72)* | 1.52 (0.79–2.93) |  |  |  |  |
| Anti-U1-RNP | 3.64 (1.09– 12.14)* | 3.62 (1.01–13.02)* |  |  |  |  |  |  |

The table demonstrates the predictors for each progressive pulmonary fibrosis (PPF) criteria. The multivariate analysis was adjusted for variables with p value < 0.1 excluding FVC, DLCO, and HRCT patterns. Data with p value < 0.05 were marked with *. Abbreviation: ATS/ERS, PPF by 2022 ATS/ERS/JRS/ALAT guideline; INBUILD, PPF by inclusion criteria of INBUILD trial; Simplified PF, PPF by previous cohort; Simplified PF + 5%, the simplified PF criteria with a threshold for HRCT ≥ 5% increase in the extent of fibrosis; OR, odds ratio; aOR, adjusted odds ratio; PVD, peripheral vascular disease; DM, diabetes mellitus; BMI, body mass index; RA, rheumatoid arthritis; SSc, systemic sclerosis; pSS, primary Sjögren's syndrome; CTD, connective tissue diseases; PFT, pulmonary function test; UIP, usual interstitial pneumonia pattern, NSIP, non-specific interstitial pneumonia pattern; ANA, antinuclear antibody.
